# Hodgkin's disease as unusual presentation of post-transplant lymphoproliferative disorder after autologous hematopoietic cell transplantation for malignant glioma

**DOI:** 10.1186/1471-2407-5-109

**Published:** 2005-08-23

**Authors:** Alberto Zambelli, Daniele Lilleri, Fausto Baldanti, Mario Scelsi, Laura Villani, Gian Antonio Da Prada

**Affiliations:** 1 U.O. di Oncologia Medica I, IRCCS, Fondazione Salvatore Maugeri, Pavia, Italy; 2 Servizio di Anatomia Patologica, IRCCS, Fondazione Salvatore Maugeri, Pavia, Italy; 3 Servizio di Virologia, IRCCS, Policlinico San Matteo, Pavia, Italy

## Abstract

**Background:**

Post-transplant lymphoproliferative disorder (PTLD) is a complication of solid organ and allogeneic hematopoietic stem cell transplantation (HSCT); following autologous HSCT only rare cases of PTLD have been reported.

Here, a case of Hodgkin's disease (HD), as unusual presentation of PTLD after autologous HSCT for malignant glioma is described.

**Case presentation:**

60-years old man affected by cerebral anaplastic astrocytoma underwent subtotal neurosurgical excision and subsequent high-dose chemotherapy followed by autologous HSCT. During the post HSCT course, cranial irradiation and corticosteroids were administered as completion of therapeutic program.

At day +105 after HSCT, the patient developed HD, nodular sclerosis type, with polymorphic HD-like skin infiltration.

**Conclusion:**

The clinical and pathological findings were consistent with the diagnosis of PTLD.

## Background

Post-transplant lymphoproliferative disorders (PTLDs) are severe complications that occur in solid organ and allogeneic hematopoietic cell transplantation (HSCT), and their incidence ranges from 1% to 20% [1].

Most PTLDs are classified as B-cell (rarely T-cell) PTLD, often related to Epstein-Barr virus (EBV) infection. Sometimes, after allogeneic HSCT, non Hodgkin lymphoma (NHL) or Hodgkin's disease (HD) have been described as PTLDs [2].

Herein, we report the first case of HD, as unusual presentation of PTLD, in a patient undergoing high dose sequential chemotherapy and autologous HSCT for malignant glioma.

## Case presentation

A 60-year-old male was diagnosed with anaplastic astrocytoma involving the left parietal and temporal lobes. The patient underwent surgical resection with subsequent magnetic resonance imaging (MRI)-proven complete excision.

In the context of a clinical trial, exploring the role of high-dose chemo-radiotherapy in malignant glioma, the patient underwent high dose sequential chemotherapy consisting of cyclophosphamide 7 g/m^2 ^plus rhG-CSF (5 μg/kbw) and hematopoietic cells harvest, followed by methotrexate 8 g/m^2 ^and thiotepa 900 mg/m^2^. Autologous unfractionated hematopoietic stem cells were administered at doses of 7.5 × 10^6^/kg CD34+ cells and the patient experienced an uncomplicated hematological recovery within 10 days.

One month later, the patient received 70-Gy of fractioned whole-brain irradiation, and steroids (dexamethasone 8 mg i.v. bid) were administered for 2 months and than tapered at the end of radiotherapy.

On day +105 he was hospitalized because of the occurrence of right hilum lymphoadenopathy associated with cutaneous lesions, as limited erythematous plaques, with well-demarcated margins and scalings.

Surgical nodes and skin excision showed the presence of HD, nodular sclerosis type, with atypical HD-like elements, infiltrating the skin. The staging evaluation, performed by CT scan of abdomen and thorax and bone marrow biopsy, was negative for distant involvement (stage IA) and no treatment was administered after radical surgery.

Eight months after HSCT, the malignant glioma relapsed and the patient received 2 cycles of carmustine (240 mg/m^2 ^i.v. q.6 weeks) without clinical benefit, followed by palliative neurosurgical subtotal excision. The steroids treatment was resumed (dexamethasone 4 mg, daily) and chronically administered as supportive care.

Six months later, histological-confirmed HD relapse occurred as bilateral hilum lymphoadenopaty. Because of deteriorated neurological clinical conditions, the patient declined any further treatment and died 2 months later for progressive glioma.

**Figure 1:**
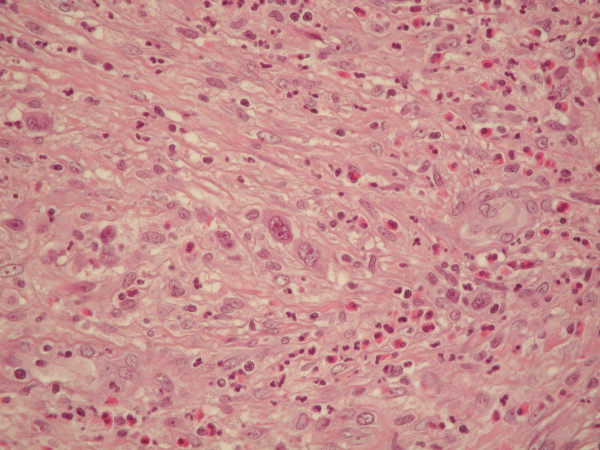
H&E stained sections of lymph node (fig.1) and skin (fig.2) excision in which the diagnostic Reed-Sternberg cell support the diagnosis of Hodgkin disease.

**Figure 2:**
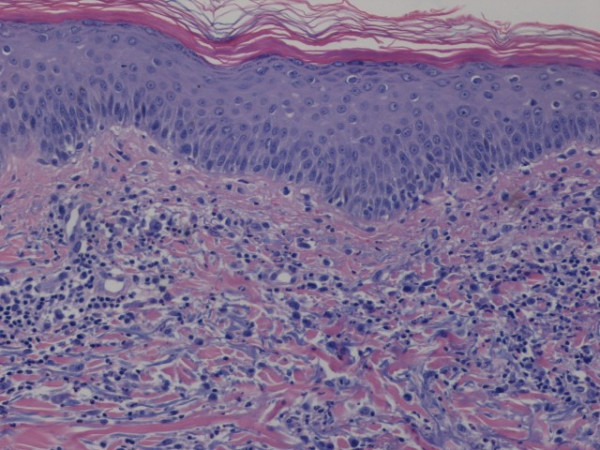
H&E stained sections of lymph node (fig.1) and skin (fig.2) excision in which the diagnostic Reed-Sternberg cell support the diagnosis of Hodgkin disease.

Lymph node and skin biopsies were fixed in 10% buffered formalin and paraffin embedded. Micron sections were then prepared and stained with hematoxylin and eosin. Paraffin section immunohistochemical stains were performed using a biotin-streptavidin technique. The lymph node structure and the skin biopsy were diffusely effaced by a growth consisting of polymorphic features of small T-cell-rich B cell lymphoma/HD-like, and typical Hodgkin's and Reed-Sternberg cells (HRSCs), nodular sclerosis type with limited areas of lymphocyte depletion (REAL) (Fig. 1 and 2). At immunohistochemistry, HRSCs expressed the following molecules: CD30+, CD43+, CD15+/-, CD3+/-; and were negative for EMA, ALK and CD20.

EBV latent membrane protein (LMP-1) was weakly positive at HRSCs immunohistochemistry, but EBV-DNA determination by PCR [3] performed in multiple node biopsy fragments and in peripheral blood mononuclear cells (PBMC) yielded negative results.

According to the clinical, histological and immunohistochemical findings, HD consistent with non EBV-associated PTLD was diagnosed.

## Conclusion

Usually reported after allogeneic transplantation, PTLD is a rare event after autologous HCT (Table 1). In most of cases, the onset is within the first in 3 months after HCT (*vs *6 months in allogeneic setting), during the time of immunosuppession.

Although HD is not the commonest form of PTLD manifestation, several reports classified HD as a possible polymorphic aspect of the post-transplant malignancies, and HD-like lesions are included among the distinctive categories of PTLD [4].

The early time of onset after HCT and the presence of risk factors for immunological impairment are considered critical for distinguishing PTLD from classic form of HD occurring later in the post-transplant period [5].

EBV appears to be the major contributor to the pathogenesis of PTLD. Uncertain is the promoting role of EBV in EBV-negative PTLDs, while a possible involvement of EBV infection in HD or T-cell PTLD appears controversial. Beside the speculative interest on the pathogenetic mechanisms of the different presentations of PTLD, EBV-negative forms of the disease pose important questions on diagnosis and treatment of PTLD.

In this case, the patient presented a post-transplant EBV-negative HD; the treatment administered for malignat glioma, consisting of immuno-suppressive procedures (HDS with autologous HCT, radiotherapy and steroids), played a role in immunologic derailment and exposed the patient to an high risk of PTLD.

In our opinion, the rapid onset of HD after HSCT (+105 day), the clinical and the histopathological characteristics are consistent with a rare presentation of HD as EBV-negative PTLD. Surveillance for PTLD should be considered also in patients affected by solid malignancies receiving autologous HSCT.

## Competing interests

The author(s) declare that they have no competing interests.

## Authors' contributions

AZ and DL equally contributed to the elaboration of the manuscript, FB performed the virological analyses, GADP performed the revision of the manuscript as responsible of the transplantation program, MS, LV performed the pathological analyses

## Pre-publication history

The pre-publication history for this paper can be accessed here:



## Figures and Tables

**Table 1:** PTLDs after autologous HCT in adults

Source (N° of cases)	Disease	HCT	PTLD (days)	PTLD (type)
Young 1989^[6] ^(2)	T cell LL	T cell purged	48/31	NHL polymorphic B cell
Chao 1993^[7] ^(1)	FML	B cell purged	52	NHL monomorphic B cell
Shephered 1995^[8] ^(1)	CML	Unmanipulated	43	NHL polymorphic B cell
Briz 1997^[9] ^(1)	T cell ALL	T cell purged	81	NHL monomorphic B cell
Hauke 1998^[10] ^(2)	HD/FLL	Unmanipulated	87/38	NHL polymorphic B cell
Peniket 1998^[11] ^(1)	MM	CD34+ selected	80	NHL monomorphic B cell
Lohrish 2000 ^[12] ^(1)	BL	Unmanipulated	39	NHL polymorphic B cell
Yufu 2000 ^[13] ^(1)	HD	Unmanipulated	900	T cell PTLD
Jenkins 2002 ^[14] ^(3)	2MM/1NHL	CD34+ selected/Unmanipulated	31/NA	NHL polymorphic B cell

ALL: acute lymphoblastic leukemia; CML: chronic myelogenous leukemia; FML: follicular mixed small cleaved and large lymphoma; FLL: follicular large cell lymphoma; LCL: large cell lymphoma; LL: lymphoblastic lymphoma; BL: Burkitt's lymphoma; HD: hodgkin disease; NHL: non hodgkin lymphoma; MM: multiple myeloma PTLD: post transplant lymphoproliferative disorder; HCT: hematopoietic cell transplantation; NA: not available.

## References

[B1] Swinnen LJ (1999). Overview of posttransplant B-cell lymphoproliferative disorders. Semin Oncol.

[B2] Deeg J, Socie G (1998). Malignancies after hematopoietic stem cell transplantation: many question, some answers. Blood.

[B3] Baldanti F, Grossi P, Furione M, Simoncini L, Sarasini A, Comoli P, Maccario R, Fiocchi R, Gerna G (2000). High levels of Epstein-Barr virus DNA in blood of solid-organ transplant recipients and their value in predicting posttransplant lymphoproliferative disorders. J Clin Microbiol.

[B4] Harris NL, Ferry JA, Swerdlow SH (1997). Posttransplant lymphoproliferative disorders: summury of Society for Hematopathology Workshop. Semin Diagn Pathol.

[B5] Rowlings PA, Curtis RE, Passweg JR, Deeg HJ, Socie G, Travis LB, Kingma DW, Jaffe ES, Sobocinski KA, Horowitz MM (1999). Increased incidence of Hodgkin's disease after allogeneic bone marrow transplantation. J Clin Oncol.

[B6] Young L, Alfieri C, Hennessy K, Evans H, O'Hara C, Anderson KC, Ritz J, Shapiro RS, Rickinson A, Kieff E (1989). Expression of Epstein-Barr virus transformation-associated genes in tissues of patients with EBV lymphoproliferative disease. N Engl J Med.

[B7] Chao NJ, Berry GJ, Advani R, Horning SJ, Weiss LM, Blume KG (1993). Epstein-Barr virus-associated lymphoproliferative disorder following autologous bone marrow transplantation for non-Hodgkin's lymphoma. Transplantation.

[B8] Shepherd JD, Gascoyne RD, Barnett MJ, Coghlan JD, Phillips GL (1995). Polyclonal Epstein-Barr virus-associated lymphoproliferative disorder following autografting for chronic myeloid leukemia. Bone Marrow Transplant.

[B9] Briz M, Fores R, Regidor C, Busto MJ, Ramon y, Cajal S, Cabrera R, Diez JL, Sanjuan I, Fernandez MN (1997). Epstein-Barr virus associated B-cell lymphoma after autologous bone marrow transplantation for T-cell acute lymphoblastic leukaemia. Br J Haematol.

[B10] Hauke RJ, Greiner TC, Smir BN, Vose JM, Tarantolo SR, Bashir RM, Bierman PJ (1998). Epstein-Barr virus-associated lymphoproliferative disorder after autologous bone marrow transplantation: report of two cases. Bone Marrow Transplant.

[B11] Lohrisch CA, Nevill TJ, Barnett MJ, Hogge DE, Connors JM, Keown PA, Gascoyne RD (2000). Development of a biologically distinct EBV-related lymphoproliferative disorder following autologous bone marrow transplantation for an EBV-negative post-renal allograft Burkitt's lymphoma. Leuk Lymphoma.

[B12] Peniket AJ, Perry AR, Williams CD, MacMillan A, Watts MJ, Isaacson PG, Goldstone AH, Linch DC (1998). A case of EBV-associated lymphoproliferative disease following high-dose therapy and CD34-purified autologous peripheral blood progenitor cell transplantation. Bone Marrow Transplant.

[B13] Yufu Y, Kimura M, Kawano R, Noguchi Y, Takatsuki H, Uike N, Ohshima K (2000). EBV associated T cell lymphoproliferative disorder following autologous blood stem cell transplantation for relapsed Hodgkin's disease. Bone Marrow Transplant.

[B14] Jenkins D, Difrancesco L, Chaudhry A, Morris D, Gluks S, Jones A, Woodman R, Brown CB, Russel J, Stewart DA (2002). Successful traetment of post-transplantation lymphoproliferative disorder in autologous blood stem cell transplant recipients. Bone Marrow Transplant.

